# Characterization of the oral microbiome of children with type 1 diabetes in the acute and chronic phases

**DOI:** 10.1080/20002297.2022.2094048

**Published:** 2022-07-11

**Authors:** Xiaoxiao Yuan, Jin Wu, Ruimin Chen, Zhihong Chen, Zhe Su, Jinwen Ni, Miaoying Zhang, Chengjun Sun, Fengwei Zhang, Yefei Liu, Junlin He, Lei Zhang, Feihong Luo, Ruirui Wang

**Affiliations:** a Department of Pediatric Endocrinology and Inherited Metabolic Diseases, Children’s Hospital of Fudan University, Shanghai, China; b Department of Pediatrics, West China Second University Hospital, Sichuan University, Chengdu, Sichuan, China; c Department of Pediatrics, Fuzhou Children’s Hospital of Fujian Medical University, Fuzhou, Fujian, China; d Department of Neuroendocrinology Pediatrics, Affiliated Hospital of Qingdao University, Qingdao, Shandong, China; e Shenzhen Children’s Hospital, Shenzhen, Guangdong, China; f Shanghai Innovation Center of TCM Health Service, Shanghai University of Traditional Chinese Medicine, Shanghai, China; g Department of Endodontics, Shanghai Stomatological Hospital & School of Stomatology, Fudan University Shanghai, China; h Department of Periodontics, Shanghai Stomatological Hospital & School of Stomatology, Fudan University, Shanghai, China

**Keywords:** Type 1 diabetes, oral microbiota dysbiosis, high-throughput sequencing, glycemic control, microbial markers

## Abstract

**Background and Aim:**

The relationship between the oral microbiota and type 1 diabetes (T1D) remains unclear. We aimed to evaluate the variations in the oral microbiome in T1D and identify potentially associated bacterial factors.

**Methods:**

We performed high-throughput sequencing of the V3-V4 area of the *16S rRNA* gene to profile the oral bacterial composition of 47 healthy children (CON group), 46 children with new-onset T1D in the acute phase (NT1D group), and 10 children with T1D in the chronic phase receiving insulin treatment (CT1D group). Multivariate statistical analysis of sequencing data was performed.

**Results:**

Compared to the CON group, the NT1D group was characterized by decreased diversity and increased abundance of genera harboring opportunistic pathogens, while this trend was partially reversed in the CT1D group. Differential genera between groups could distinguish the NT1D group from the CON group (AUC = 0.933) and CT1D group (AUC = 0.846), respectively. Moreover, T1D-enriched genera were closely correlated with HbA1c, FBG and WBCs levels.

**Conclusion:**

Our results showed that the acute phase of T1D was characterized by oral microbiota dysbiosis, which could be partially ameliorated via glycemic control. The possible role of oral microbiota dysbiosis on oral health and systemic metabolic status in T1D warrants further mechanistic investigation.

## Graphical abstract

**Figure:**
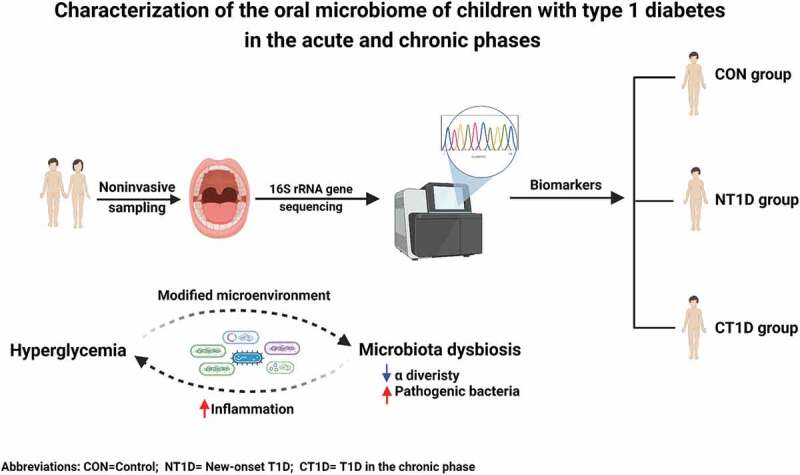

## Introduction

Type 1 diabetes (T1D) is a pediatric-onset disease that results in chronic insulin deficiency and consequent hyperglycemia. Clinically, the new onset of T1D follows an acute phase in most cases and may be accompanied by acute life-threatening complications, such as diabetic ketoacidosis. Due to the progressive nature of T1D, in the chronic phase, patients require life-long insulin therapy to maintain euglycemia, and poor glycemic control may result in serious long-term chronic complications, including periodontal diseases.

The oral microbiota, which comprises over 700 bacterial species, is considered the second most complex bacterial community in the human body [1]. The oral microbiome constantly interacts with the human body and varies in response to oral and systemic diseases [2–5]. For example, high glucose content in oral fluids contributes to the proliferation of genera harboring many opportunistic pathogens, such as *Streptococcus*, and increases the risk of periodontal diseases [6,7]. Furthermore, a bidirectional relationship has been proposed between oral health and systemic diseases [8]. Dysbiosis of the oral microbiome has been observed in many oral diseases and whole-body systemic diseases, such as periodontal diseases, diabetes mellitus, cardiovascular diseases, and tumors [4]. In return, oral microbiota dysbiosis may aggravate these diseases by inducing local and systemic inflammation.

Oral health issues, which are highly associated with dysbiosis of the oral bacterial community in patients with T1D and T2D, are increasingly attracting attention. Reportedly, the prevalence of periodontitis among T1D teenagers shows a 5-fold increase, compared to that of the controls [9]. Similar to those of patients with T2D, the oral microbiota of T1D patients too were markedly different from that of healthy controls, characterized by an increased abundance of genera harboring many opportunistic pathogens, such as *Streptococcus, Actinomyces, Rothia*, and *Brevundimonas* [10,11]. According to some studies, patients with a better-regulated T2D displayed a significantly lower abundance of disease-associated oral bacterial genera [12] as well as a lower prevalence of severe periodontitis [13] than did patients whose diabetes was poorly regulated, thereby supporting an association between better-regulated diabetes and milder oral microbiota dysbiosis and oral diseases. In addition, the degree of chronic hyperglycemia in T1D is positively related to the severity of periodontal diseases. However, in-depth analyses of oral bacterial communities are lacking [14]. Whether there exist structural changes in the oral microbiota at different phases of type 1 diabetes has not been reported. In this study, we aimed to delineate the community structure of the oral microbiome in T1D children in both the acute and chronic phases and to explore the association with systemic metabolic status.

## Materials and methods

### Study population

This cross-sectional study was conducted at the Children’s Hospital of Fudan University in China. In total, 111 participants were recruited. After excluding ineligible subjects (based on exclusion criteria), as well as those who were unable to complete the study, 46 children with new-onset T1D, who were first diagnosed with T1D according to the criteria of the American Diabetes Association [15], 10 children with T1D in the chronic phase receiving insulin treatment, and 47 healthy children between January 2020 and July 2020, remained in the study. Healthy subjects visited the outpatient clinic of the hospital for routine medical checkups. The exclusion criteria pertinent to this study were dental caries, dental erosion, periodontal diseases or other oral diseases, acute or chronic inflammatory diseases or infectious diseases, other severe organic lesions and metabolic diseases, or having received antibiotics/probiotics/prebiotics/or any other medical treatment within 1 month. Participant characteristics are summarized (Supplementary Table S1). The age-stratified analysis focused on three age subgroups: low-age subgroup (3 ≤ age ≤ 7), middle-age subgroup (7 < age ≤ 11), and high-age subgroup (age >11). Written informed consent was obtained from all study participants before sample collection, and the study was approved by the Institutional Review Board and Ethics Committee of Children’s Hospital of the Fudan University ([2019]210.

### Saliva sample collection

Saliva samples were collected before breakfast (approximately 7:00 h), under the guidance of trained physicians. Participants were required to fast for 10 h and to avoid brushing their teeth, as well as eating, drinking, or smoking. Physicians drawing samples cleaned their hands thoroughly before wearing gloves. Participants also washed their hands and performed other necessary cleaning measures. Ten minutes before sampling, the participants washed their oral cavity with sterile distilled water. Each participant was required to retain saliva in their mouth for at least 1 min, before collecting the saliva sample using a microcentrifuge tube. The collection containers delivered to the participants were labeled with their names and sampling dates. To ensure that sufficient saliva was collected (approximately 1 mL), this process was repeated several times. All samples were immediately transferred to the laboratory within 10 min and stored at −80°C until DNA extraction for amplification and sequencing.

### Blood collection and laboratory measurements

Peripheral venous blood (3 mL) was drawn in the morning after 10 h of fasting. Blood samples were centrifuged at 3,000 rpm for 10 min at 4°C. Blood hemoglobin A1c (HbA1c), fasting blood glucose (FBG), C peptide, white blood cells (WBCs), cholesterol (TC), triglycerides (TG), high-density lipoprotein (HDL), and low-density lipoprotein (LDL) levels were measured according to standardized procedures and uniform national quality control protocols.  

### DNA extraction and PCR amplification

Microbial DNA was extracted from saliva samples using an E.Z.N.A.® soil DNA Kit (Omega Bio-tek, Norcross, GA), according to the manufacturer’s protocols. The DNA extract was analyzed on a 1% agarose gel, following which DNA concentration and purity were determined using a NanoDrop 2000 UV-vis spectrophotometer (Thermo Scientific, Wilmington, DE). The hypervariable region V3-V4 of bacterial *16S rRNA* was amplified using the primer pairs 338 F (5’-ACTCCTACGGGAGGCAGCAG-3’) and 806 R (5’-GGACTACHVGGGTWTCTAAT-3’) on an ABI GeneAmp® 9700 PCR thermocycler (ABI, CA). PCR amplification of the *16S rRNA* was performed as follows: initial denaturation at 95°C for 3 min; followed by 27 cycles of denaturation at 95°C for 30s; annealing at 55°C for 30s; extension at 72°C for 45s; single extension at 72°C for 10 min; and ending at 4°C. The PCR mixtures contained 4 μL of 5× TransStart FastPfu buffer, 2 μL of 2.5 mM dNTPs, 0.8 μL forward primer (5 μM), 0.8 μL reverse primer (5 μM), 0.4 μL TransStart FastPfu DNA Polymerase, 10 ng template DNA, and finally up to 20 μL ddH_2_O. PCR was performed in triplicate. The PCR product was extracted from 2% agarose gel and purified using an AxyPrep DNA Gel Extraction Kit (Axygen Biosciences, Union City, CA), according to the manufacturer’s instructions, and quantified using a Quantus™ Fluorometer (Promega, WI).

### Illumina MiSeq sequencing

NGS library preparations and Illumina MiSeq sequencing were performed at Shanghai Honsun Biological Technology Co., Ltd. (Shanghai, China). Raw reads were deposited in the NCBI Sequence Read Archive database.

### Processing of sequencing data

Raw *16S rRNA* sequencing reads were quality-filtered using the fastp version 0.20.0 and merged by the FLASH version 1.2.11 [16] using the following criteria: (i) reads (300 bp) were truncated at sites receiving an average quality score of < 20 over a 50 bp sliding window, and truncated reads shorter than 50 bp as well as reads containing ambiguous characters were discarded; and (ii) only overlapping sequences longer than 10 bp were assembled according to their overlapping sequence, where the maximum allowed mismatch ratio in the overlapping region was 0.2 and reads that could not be assembled were discarded; and (iii) samples were distinguished according to the barcode and primers, and sequence direction was adjusted for exact barcode matching, with two nucleotide mismatches in primer matching. Operational taxonomic units (OTUs) with a 97% similarity cut-off were clustered using the UPARSE version 7.1 [17], while chimeric sequences were identified and removed. The taxonomy of each OTU representative sequence was analyzed using the RDP Classifier version 2.2 [18] against the 16S rRNA database using a confidence threshold of 0.7.

### Statistical analyses

Student’s t-test or Wilcoxon rank-sum test was used to detect variations in clinical parameters, depending on the data distribution. The chi-squared test was applied to categorical variables. Spearman’s correlation analysis was used for correlation analysis. Principal coordinate analysis (PCoA) was used to compare changes among study groups. Differences in diversity and taxonomic composition were evaluated using Kruskal–Wallis or Wilcoxon rank-sum tests. The most discriminatory bacteria among the groups were identified using the linear discriminant analysis (LDA) effect size (LEfSe) method. Data processing and statistical analyses were performed using SPSS version 21, GraphPad Prism version 8, and R version 3.5.1. *P < 0.05; **P < 0.01; ***P < 0.001.

## Results

### Anthropometric and biochemical measurements of study participants

**Figure 1.**
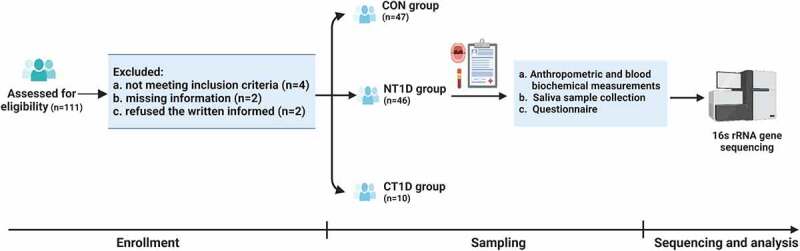
Flow chart illustrating the procedures of the study. Created with Biorender.com.

A total of 47 healthy children (CON group) and 46 children with the new-onset T1D (NT1D group), who underwent a strict pathological diagnosis and satisfied the requirements of the exclusion process, were enrolled (Figure 1). Another 10 T1D children in the chronic phase who received insulin treatment (CT1D group) were also enrolled for further comparative analysis. Patient characteristics, including age, sex, and BMI, were balanced between the CON and NT1D groups and between the NT1D and CT1D groups, respectively. The NT1D group had low C-peptide levels and high fasting blood glucose and HbA1c levels. Compared with the CON group, the WBC count was significantly increased in the NT1D group. In addition, the NT1D group showed a significant decrease in HDL levels and a significant increase in TG levels. The CT1D group had received insulin therapy for 2.57 years on average. HDL levels of the CT1D group showed a significant increase compared with that of the NT1D group. No other significant differences were observed between the groups. Detailed demographic statistics and clinical parameters of the study participants are shown (Supplementary Table S1).

### Significant variation of the oral microbiota profiles in T1D

**Figure 2.**
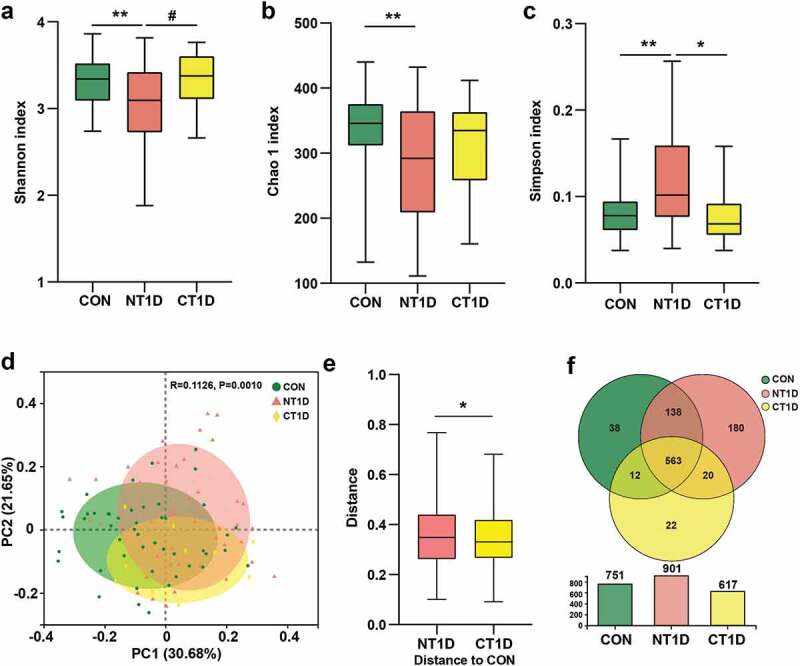
Taxonomic profiles of oral microbial communities in T1D. **(a-c)** Microbial community richness (Chao 1) and diversity (Shannon and Simpson). **(d)** PCoA analysis of three groups based on the Bray-Curtis distance. **(e)** Distance compared to the CON group through the Anosim algorithm. **(f)** Venn diagram showing the overlap of OTUs between groups. Data were expressed as mean ± S.E.M. # 0.05 < P < 0.1, *P < 0.05, **P < 0.01.

To investigate the composition of the oral microbiota, we sequenced the V3-V4 region of the *16S ribosomal RNA* gene in saliva samples. In total, 7,946,410 high-quality *16S rRNA* gene sequences were identified. After taxonomic assignment, a total of 972 OTUs were identified. The species accumulation curve of all samples successfully reached an asymptote, supporting the adequacy of our sampling efforts (Supplementary Figure S1a). A rank abundance curve was generated to further demonstrate species abundance and evenness (Supplementary Figure S1b). The α diversity indexes were assessed according to richness (Chao 1) and diversity (Shannon and Simpson), wherein a high Shannon index and low Simpson index represented high community diversity. Compared to the CON group, the NT1D group was characterized by a significant drop in Chao 1 and Shannon indexes and an increase in the Simpson index, while the CT1D group showed a recovery trend towards the CON group (Figure 2(a-c)). β diversity based on the Bray-Curtis distance indicated significant differences between the compositions of the oral microbiomes among groups (Figure 2(d)). The distance between the CT1D and CON groups was significantly shorter than that between the NT1D and CON groups (Figure 2(e)). In the age-stratified analysis (Supplementary Figure S2), microbial α diversity of the NT1D group at different age subgroups was significantly decreased compared to that of the CON group, while this trend was partially reversed in the CT1D group. A significant difference in β diversity was also observed among the three groups in the low-age subgroup analysis. There is no significant difference among the three groups in the middle- and high-age subgroup analysis which may be due to the sample size of the subgroup. Moreover, the Venn diagram showed that 563 of the 973 OTUs were shared among these three groups, where 180 OTUs were unique to the NT1D group, and 22 were specific for CT1D (Figure 2(f)). The above results suggested that the community diversity had been considerably decreased and that the structure of the microbial communities in the NT1D group had been altered, while the CT1D group had partially recovered from oral microbiota dysbiosis.

### Taxonomic profiles of the microbial communities in T1D

**Figure 3.**
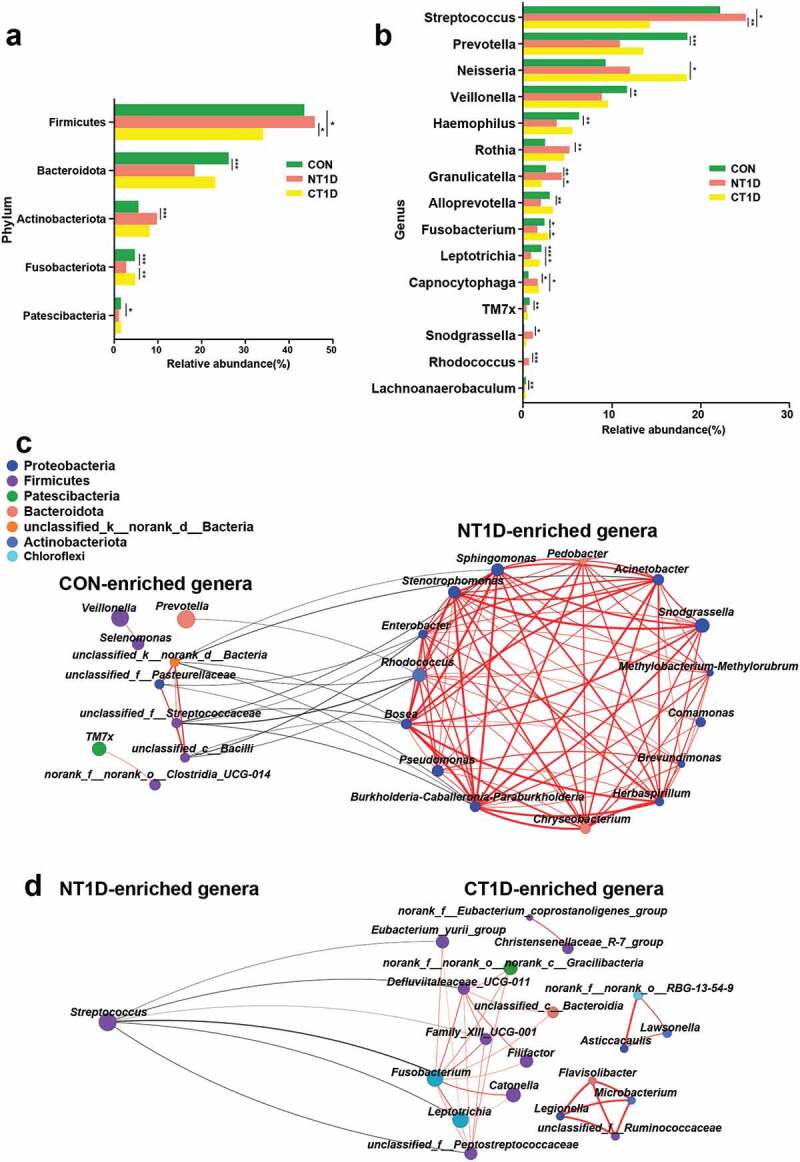
Differentiated bacteria between groups and network analysis. **(a)** Bacterial composition at the phylum level. **(b)** Bacterial composition at the genus level. **(c,d)** Network analysis of differential bacteria obtained from the Wilcoxon rank-sum test with positive interactions in red and negative interactions in black. *P < 0.05, **P < 0.01, ***P < 0.001.

To further identify differentially abundant bacterial taxa among the three groups, we analyzed the relative abundance of bacterial communities at the phylum and genus levels. A strong microbial imbalance at different taxonomic levels was observed in the NT1D group compared with that in the CON group. For all phenotypes, the dominant phyla were Firmicutes, Bacteroidota, Proteobacteria, Actinobacteriota, and Fusobacteriota. The NT1D group was characterized by a significantly higher abundance of Firmicutes and Actinobacteriota and a lower abundance of Bacteroidota and Fusobacteriota compared to the CON group, with the community structure of the CT1D group showing an opposite trend compared to the NT1D group (Figure 3(a)). A comparison of microbial abundance at the genus level revealed that the abundance of dominant genera harboring many opportunistic pathogens, such as *Streptococcus* (FC = 1.13), *Granulicatella* (FC = 1.68), *Rothia* (FC = 2.07), and *Rhodococcus* (FC = 4.44), in the NT1D group were significantly higher than those in the CON group, while the CT1D group showed lower abundance (Figure 3(b) and Supplementary Figure S3). Compared to that of the CON group, the abundance of *Veillonella* (FC = 0.76) and *Prevotella* (FC = 0.59) in the NT1D group was decreased, whereas that in the CT1D group showed a decreasing trend. Interestingly, the abundance of genera harboring pathogenic bacteria, such as *Neisseria*, and potential periodontal pathogens, such as *Capnocytophaga*, in both the NT1D and CT1D groups were consistently high compared to that in the CON group. Network analysis indicated that genera, such as *Enterobacter, Rhodococcus, Sphingomonas* and *Stenotrophomonas*, that were enriched in the NT1D group, as compared with those in the CON group, were positively correlated and formed a close cluster. These genera were negatively correlated with those enriched in the CON group (Figure 3(c)). *Streptococcus*, the genus enriched in the NT1D group, compared to the CT1D group, was negatively correlated with six genera enriched in the CT1D group including *Fusobacterium, Leptotrichia*, the *Eubacterium_yurii_group*, *Defluviitaleaceae_UCG-011*, *Family_XIII_UCG-001*, and *unclassified_f__Peptostreptococcaceae* (Figure 3(d)).

### Oral microbiota-based biomarkers for T1D

Next, we applied the LEfSe method to analyze specific differences between the compositions of microbiotas and to search for potential biomarkers among these three groups. The cladogram represents significantly different taxa among the three groups according to a hierarchy that reflects the taxonomic rank from phylum to genus (Supplementary Figure S5), which was consistent with the whole oral microbiota structure (Figure 3). *Leptotrichia*, a health-associated genus; *TM7x*, which virulently kills host bacteria; and *Prevotella*, an acetate-producing bacterium; were significantly enriched in the CON group. Potential periodontal pathogens, such as *Capnocytophaga* and *Granulicatella*, and genera harboring many opportunistic pathogens, such as *Streptococcus, Staphylococcus*, and *Enterobacterales* were significantly enriched in the NT1D group. *Fusobacterium, Leptotrichia*, and *Eubacterium* were found to be significantly enriched in the CT1D group. Thus, these bacteria could be considered hyperglycemia-associated taxa and potential biomarkers for T1D.

**Figure 4.**
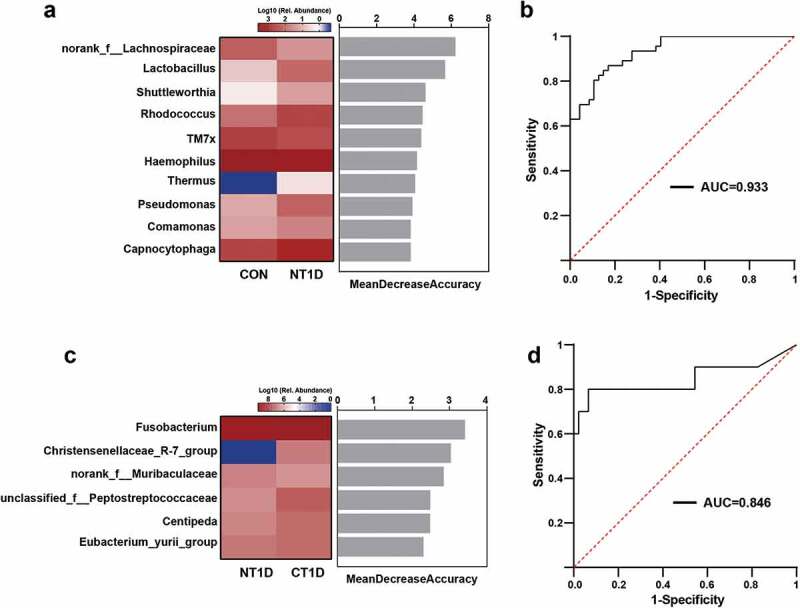
Oral microbiota-based biomarkers for T1D based on a random forest classification model. **(a,c)** Classification performance of the most discriminant genera by random forest analysis and heatmap based on the relative abundance of the genera. **(b,d)** Receiver operating characteristic curves and their corresponding AUCs.

Furthermore, a random forest classifier model was constructed using the area under the curve (AUC)-RF algorithm. As a result, 10 genera ranked by *norank_f__Lachnospiraceae* and *Lactobacillus* were selected as the key bacteria of T1D, achieving an AUC value of 0.933 (Figures 4(a,b)). Six markers, including *Fusobacterium* and *norank_f__Muribaculaceae*, were selected as the optimal marker set that could be used to distinguish the CT1D group from the NT1D group, with an AUC of 0.846 (Figure 4(c,d)). Our findings suggested that the oral microbiota could help distinguish individuals with T1D from healthy controls, thereby providing some suggestive evidence for adjuvant diagnosis and treatment of T1D by noninvasive means.

### Associations between oral bacteria and clinical indicators

**Figure 5.**
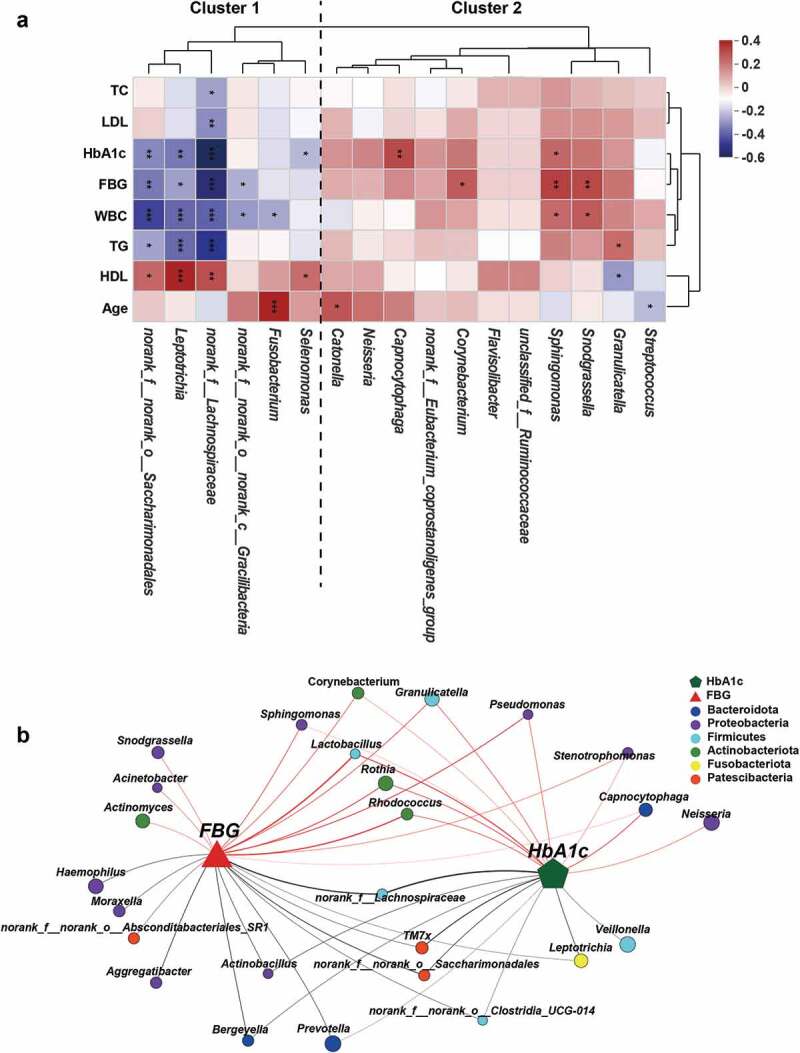
Associations of the oral microbial genera with clinical indicators. **(a)** Heatmap of the Spearman’s correlation between clinical indicators and discriminatory genus. Red squares indicate positive correlations, whereas blue squares indicate negative correlations. **(b)** Correlation network between FBG, HbA1c, and the top 50 high-abundant genera. Correlations were identified by Spearman’s rank correlation coefficient > 0.60 and P < 0.05. CON-enriched genera were presented below, while NT1D-enriched genera were presented above. *P < 0.05, **P < 0.01, ***P < 0.001.

Our results demonstrated evident oral microbiota dysbiosis, characterized by significant compositional changes in T1D. To further evaluate whether T1D-associated clinical parameters were related to changes observed in the oral microbiota, we performed a Spearman rank correlation analysis to determine the degree of association between clinical parameters and the oral microbiota at the phylum and genus levels. Key genera were divided into two clusters. Cluster 2 mainly consisted of genera harboring opportunistic pathogens, such as *Granulicatella, Sphingomonas*, and *Streptococcus*, while cluster 1 consisted of some genera, such as *norank_f_Lachnospiraceae* and *Leptotrichia*, which showed a higher abundance among healthy individuals (Figure 5(a)). Indicators of glycolipid metabolism, such as HbA1c, FBG, TG, and LDL, were negatively correlated with genera in cluster 1 and positively correlated with genera in cluster 2, whereas HDL showed the opposite trend. WBC, an index for systemic inflammation, was inversely related to cluster 1 but positively related to cluster 2. In addition, *Streptococcus* showed a significantly negative correlation with age, while *Fusobacterium* and *Catonella* showed a significantly positive correlation. At the phylum level, Actinobacteriota displayed a strong positive association with HbA1c, FBG, WBCs, and TG, while Bacteroidota, Fusobacteriota, and Patescibacteria showed a significantly negative correlation with the above indexes (Supplementary Figure S7). Interestingly, both FBG and HbA1c were significantly positively associated with some potential opportunistic and periodontal pathogens, represented by *Pseudomonas, Granulicatella, Rothia*, and *Rhodococcus*, and negatively associated with CON-enriched genera, represented by *Lachnospiraceae*, *TM7x*, and *Prevotella* (Figure 5(b)), indicating that these genera may constitute potential biomarkers linking oral microbiota and metabolic status. The broad correlation between metabolic parameters and key bacteria indicates that the oral microbiota may be associated with systematic glucose metabolism, lipid metabolism, and inflammation response in T1D.

## Discussion

Accumulating evidence has highlighted oral health issues in diabetes, which are highly associated with oral bacterial dysbiosis. However, in-depth characterization of the oral bacterial community and structural alterations in the oral microbiota at different phases of T1D remains scarce. For the first time, our study has revealed oral microbial structures and signatures associated with acute as well as chronic phases of childhood T1D. Dysbiosis of the oral microbiota in the acute phase of T1D is characterized by reduced diversity and a perturbed structure, such as an increased abundance of genera harboring many opportunistic pathogens, which may be alleviated via glycemic control in the chronic phase.

In this study, we found that the NT1D group without oral diseases was characterized by oral microflora dysbiosis, as shown by a decrease in microbial diversity and an increase in the abundance of genera harboring many opportunistic pathogens [19,20], such as *Streptococcus, Granulicatella, Rothia*, and *Rhodococcus*, which are associated with infectious processes and periodontitis and may increase the future risk of oral diseases. Interestingly, we found that the CT1D group, which received insulin treatment, showed partial recovery from dysbacteriosis. Although no difference in HbA1c was observed between the two diabetic groups, blood glucose in the CT1D group, during ordinary daily life, may have been more stable than that in the N1TD group. Our study suggests that glycemic control may reduce long-term complications of diabetes in various organ systems, such as oral diseases. Poor periodontal health is prevalent in T1D [**21**,^22^]. Individuals with T1D and healthcare providers should initiate lifestyle as well as medical interventions to reduce oral health issues [23]. Adequate metabolic control may decelerate the proliferation of pathogenic oral bacteria. Although it is unclear whether the association is causative or reactive, the findings of this study indicate the usefulness of interventions, such as the manipulation of the oral microbiota, especially in high-risk populations.

While exploring specific changes in the oral bacterial composition, we found that the NT1D group had a lower α diversity and a significant separation of microbiomes compared to the CON group, suggesting reduced stability of the oral bacterial community in the acute phase of T1D, which was consistent with reports indicating that T2D adolescents exhibited decreased oral bacterial diversity [24]. The age-stratified analysis demonstrated a significant difference between α and β diversity among the three groups. This is consistent with the results of our analysis, in general, which indicated that the differences in the overall bacterial community stabilized with age. The abundance of the phylum Firmicutes, which is positively associated with periodontitis and insulin resistance [25], was increased in the NT1D group and decreased in the C1TD group. We further characterized disease-specific changes in oral bacterial structures, such as an increase in the genera *Streptococcus, Granulicatella, Rothia*, and *Rhodococcus*, and a decrease in the genera *Prevotella, Veillonella*, and *Leptotrichia* in the NT1D group, while the CT1D group showed a decreasing trend. Moreover, HbA1c, FBG, TG, and WBCs were positively correlated with the above-mentioned T1D-enriched bacteria and negatively correlated with the decreased microbiota in T1D. Oral streptococci, especially *Streptococcus mutans* and *Streptococcus sobrinus*, are generally considered primary etiologic agents of dental caries in humans [26]. *Granulicatella* spp. and *Rothia* spp. have been reported as potential periodontal pathogens [27,28]. Previous clinical and animal studies have further demonstrated that increased oral microbial pathogenicity in diabetes is linked to increased inflammation and IL-17A expression [29,30]. Considered together, oral microbiota dysbiosis in diabetes may not only represent a typical feature of hyperglycemia but might also be closely related to disease progression.

The oral microbiota plays a crucial role in maintaining oral homeostasis and systemic health [31]. Early identification and intervention in oral dysbiosis are crucial for not only the prevention of oral diseases but also the management of systemic diseases [32]. In the exploration of oral microbiota-targeted biomarkers, a study found that the oral microbial prediction model could yield excellent discriminating performance of periodontitis patients from healthy individuals, with the accuracy for test achieving 90% [33]. In our study, oral microbial markers identified by random forest models could effectively distinguish the NT1D group from the CON group (AUC = 0.933) and CT1D group (AUC = 0.846), respectively. Our study, together with previous studies, suggests that the oral microbiota could potentially serve as non-invasive biomarkers for T1D adjuvant assessment of glycemic control and oral health.

Nevertheless, this study was affected by some limitations. Firstly, further studies using larger samples of T1D children in the chronic phase may be needed to confirm the results of this study. Secondly, the cross-sectional nature of the study did not enable a better understanding of the mechanisms and time sequences linked to the observed associations. Further exploration aimed at finding whether variation in bacterial abundance is a causative factor of T1D or a derivational reflection of T1D may be needed. Thirdly, the key differential microbiotas identified by our study were somewhat different from those reported by a previous study [11]. Such discrepancies may be attributed to racial differences between studies or the progressive nature of T1D, suggesting that microbiota-based biomarkers are ethnicity-dependent and should be validated in a wide range of populations.

## Conclusion

Our study is the first to profile the overall structural changes of oral microbiota communities at different phases of T1D. Potential opportunistic pathogens enriched in the acute phase of T1D are positively related to increased inflammation and disordered glycolipid metabolism. Oral microbiota dysbiosis in the chronic phase of T1D is partially ameliorated with insulin therapy, indicating that glycemic control may ameliorate oral microbiota disturbance and oral complications. Our study could provide significant insights for solving oral health problems in T1D, while the underlying mechanism and pathogenic roles of oral microbiota dysbiosis need to be studied in the future.

## Supplementary Material

Supplemental Material

## Data Availability

Data of the present study were presented in this article. Additional datasets, if required, can be provided by the corresponding authors on reasonable request.
