# Supplementary material for: Characterization of the oral microbiome of children with type 1 diabetes in the acute and chronic phases
Source: J Oral Microbiol. 2022 Jul 11;14(1):2094048. doi: 10.1080/20002297.2022.2094048 (PMC9291685; doi:10.1080/20002297.2022.2094048)
Supplement: Supplemental Material [file ZJOM_A_2094048_SM7895.doc]

**Characterization of the oral microbiome of children with type 1 diabetes in the acute and chronic phases**

| **Parameters** | **CON group** | **NT1D group** | **CT1D group** |
| --- | --- | --- | --- |
| Female, n (%) | 19 (40.43) | 24 (52.17) | 5 (50.00) |
| Age (years) | 7.5±3.0 | 8.3±3.0 | 10.4±3.9 |
| BMI | 15.9±1.8 | 16.2±2.5 | 15.6±2.6 |
| HbA1c (%) | 5.07±0.31 | 12.91±2.39 *** | 13.35±2.31 |
| FBG (mmol/L) | 5.34±0.37 | 23.31±8.90 *** | 19.35±4.70 |
| C peptide (ng/ml) | ND | 0.36±0.34 | 0.22±0.14 |
| WBC (x10^9/L) | 5.74±1.41 | 8.96±5.23 *** | 11.62±14.25 |
| TC (mmol/L) | 4.72±0.89 | 4.95±1.45 | 4.88±0.97 |
| TG (mmol/L) | 0.71±0.34 | 1.52±1.71 *** | 1.63±1.22 |
| HDL (mmol/L) | 1.52±0.30 | 1.28±0.40 *** | 1.56±0.45 ^#^ |
| LDL (mmol/L) | 2.66±0.57 | 3.08±1.16 | 2.67±0.75 |
| Insulin treatment (years) | 0 | 0 | 2.57 |

**Supplementary Table 1 Characteristics of study participants.** Data are expressed in mean ± SD. P value: * NT1D vs. CON; # CT1D vs. NT1D. * P < 0.05, ** P < 0.01, *** P < 0.001, # P < 0.05. ND, not determined; BMI, body mass index; HbA1c, hemoglobin A1c; FBG, fasting blood glucose; WBC, white blood cell; TC, cholesterol; TG, triglycerides; HDL, high-density lipoprotein; LDC, low-density lipoprotein.

**
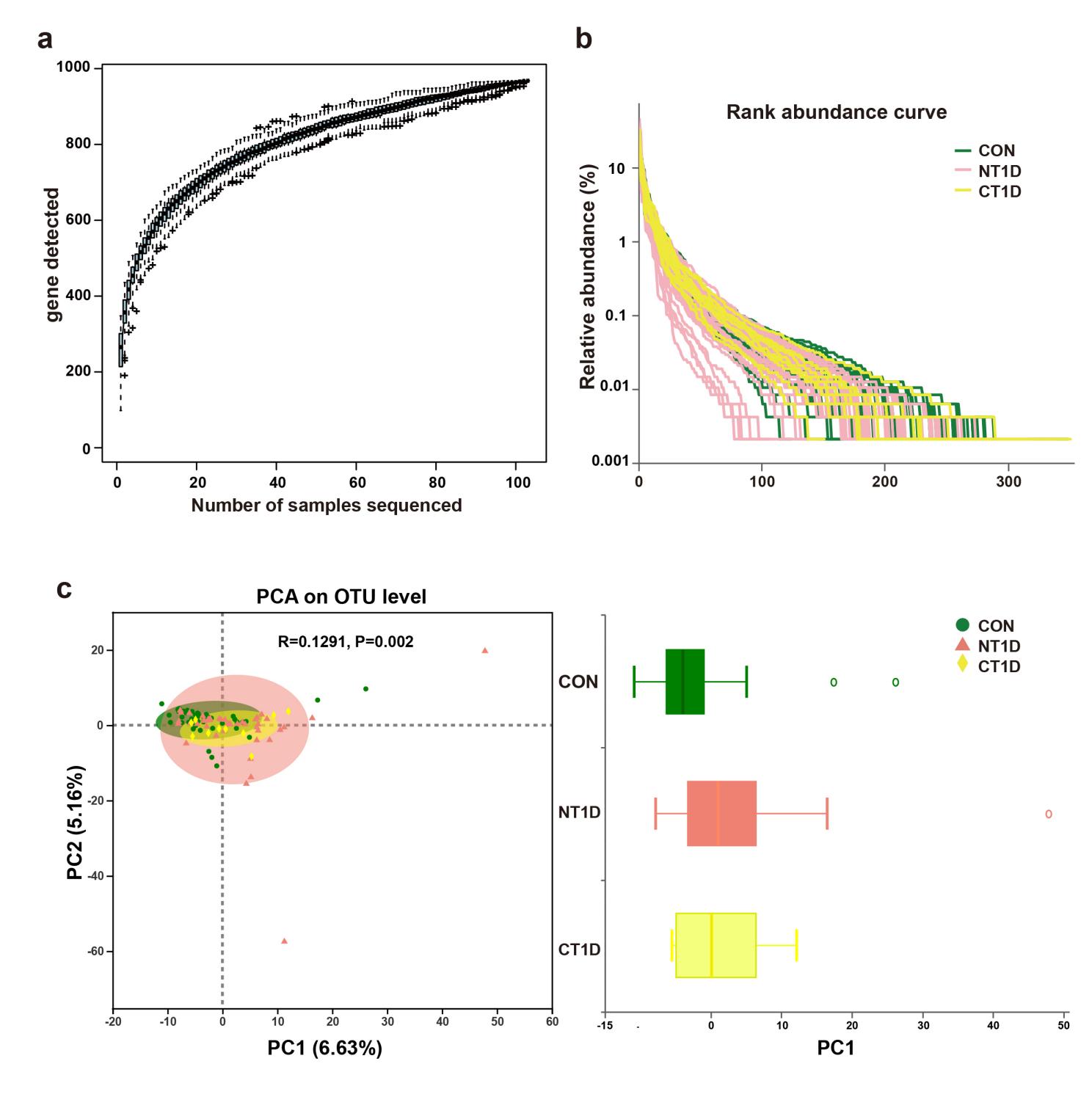
**

**Figure S1** (a) Species accumulation curve. (b) Rank abundance curve of sequencing samples. (c) PCA plot of PC1 versus PC2.


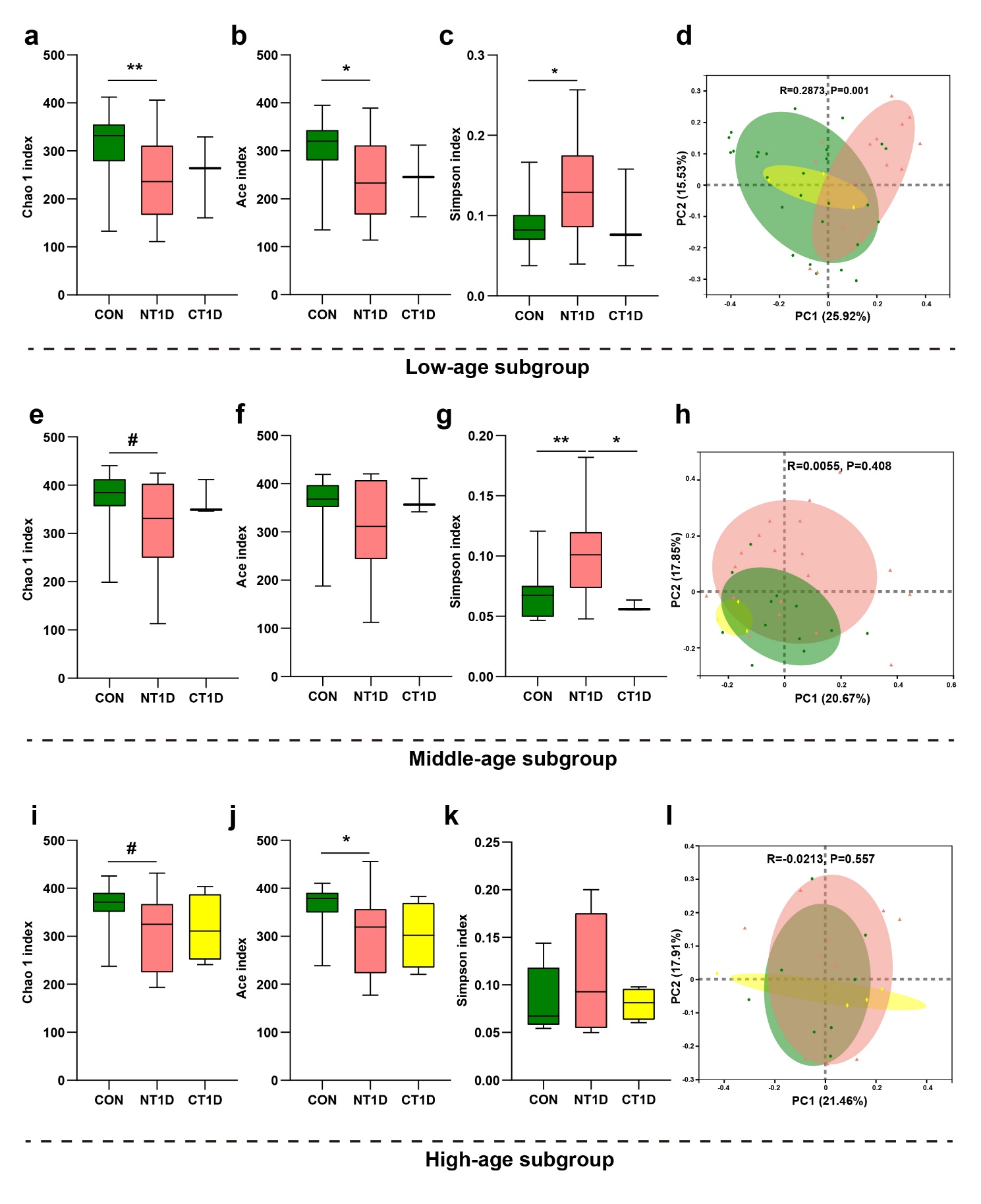
**Figure S2**  α and β diversity analyses in different age subgroups. (a-c, e-g, i-k) Microbial community richness (Chao 1 and Ace) and diversity (Simpson). (d, h, l) PCoA of three groups based on the Bray-Curtis distance.


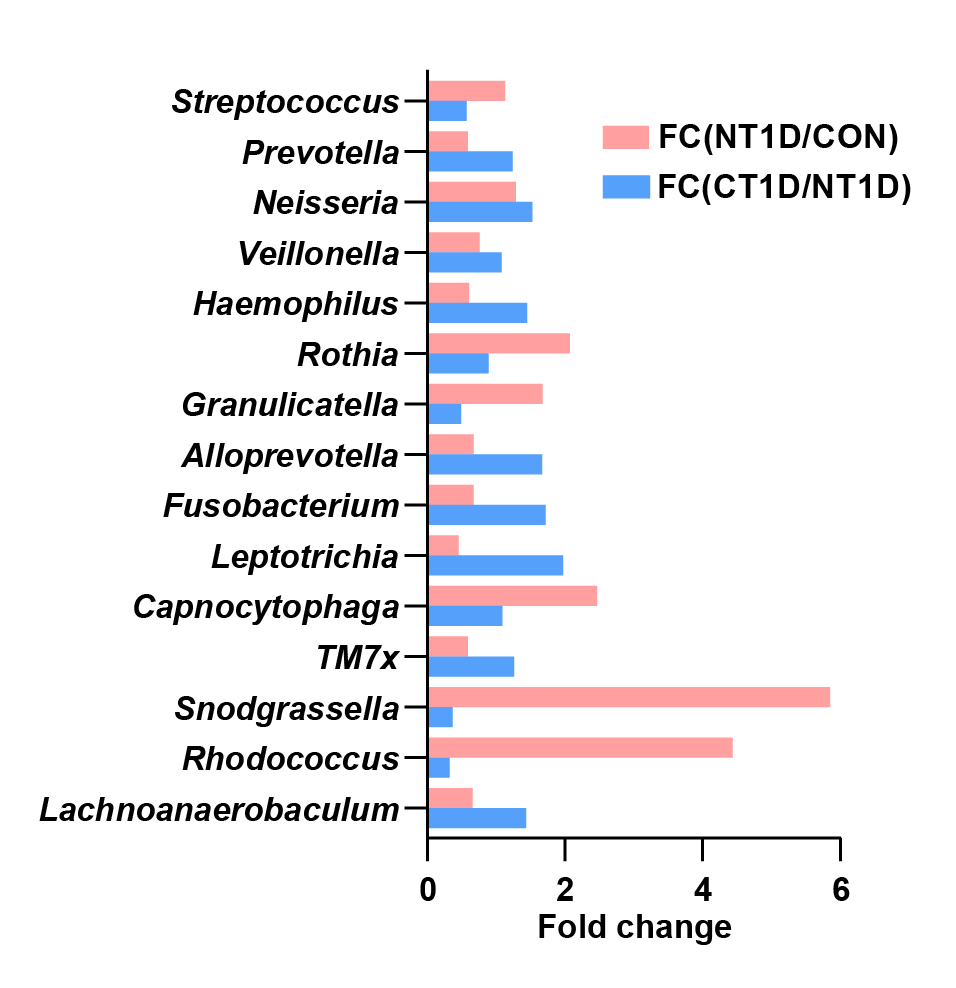


**Figure S3** Foldchange (FC) plot showing the top 15 genera with differential abundance. The FC value was calculated via the ratio of the relative abundances between the two groups.

**
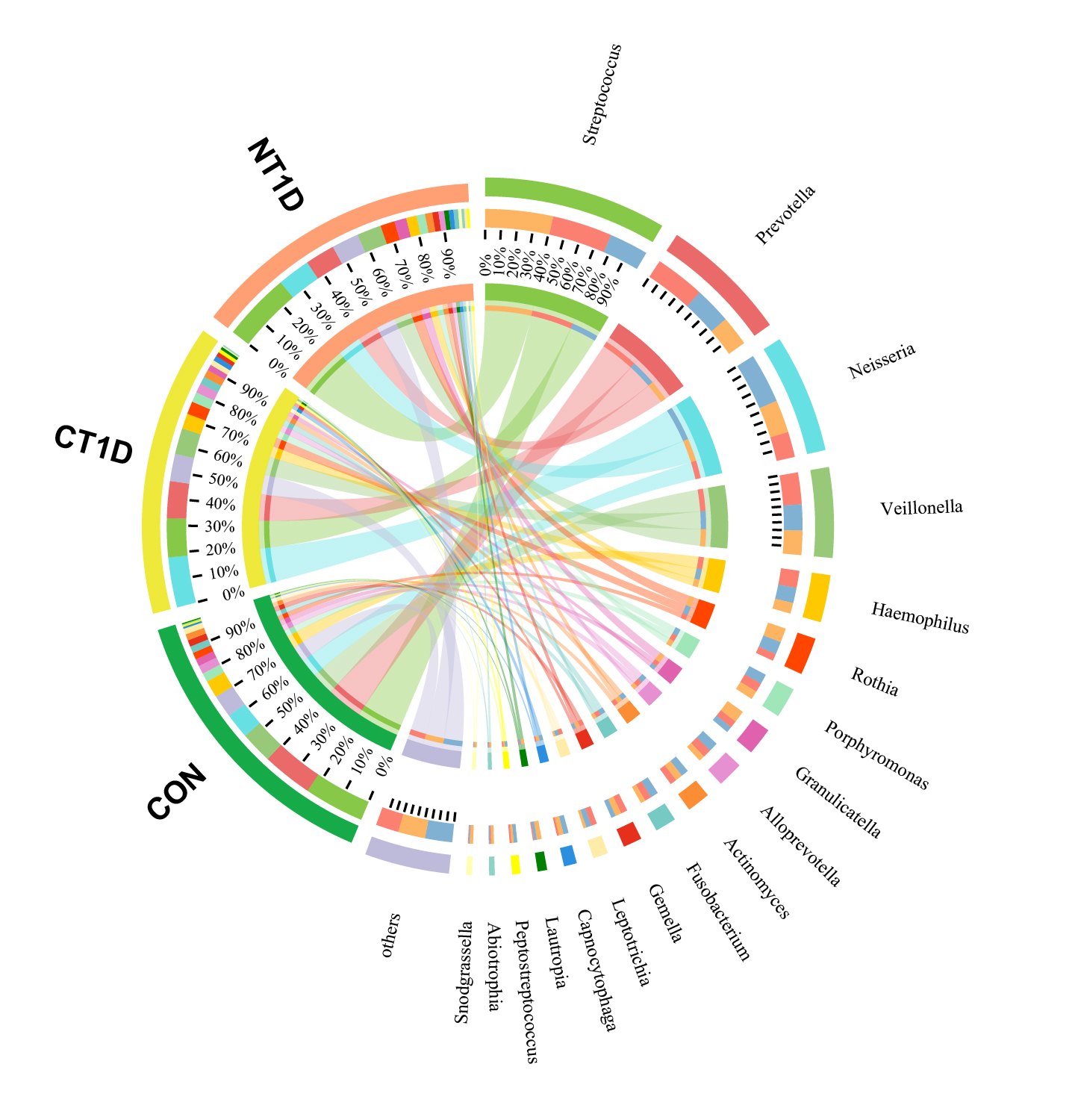
**

**Figure S4** For the gut microbiota data analysis, the distribution of microbial community among three groups was visualized by Circos at the genus level.


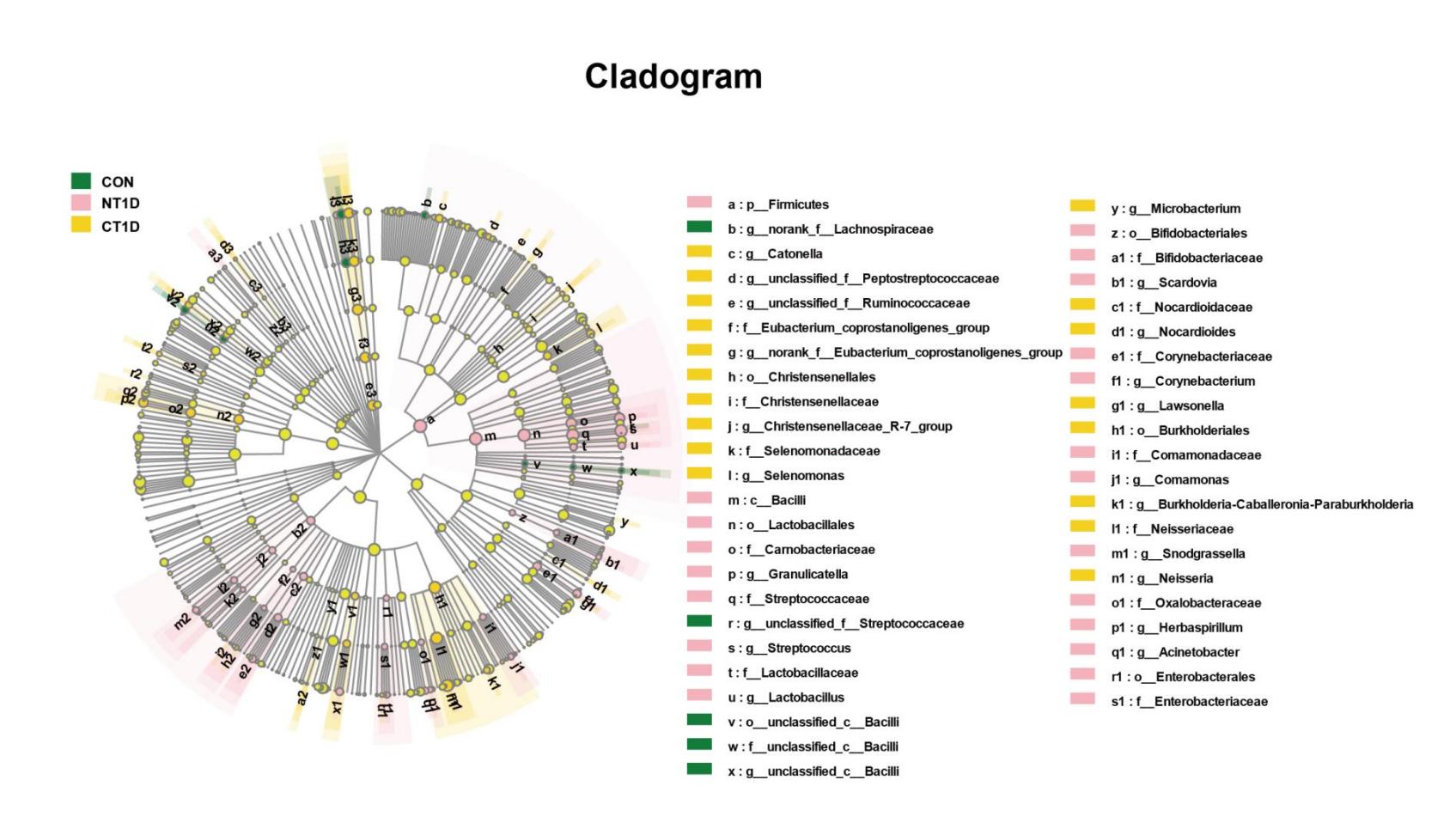


**Figure S5** LEfSe taxonomic cladogram generated from 16S rRNA gene sequences.


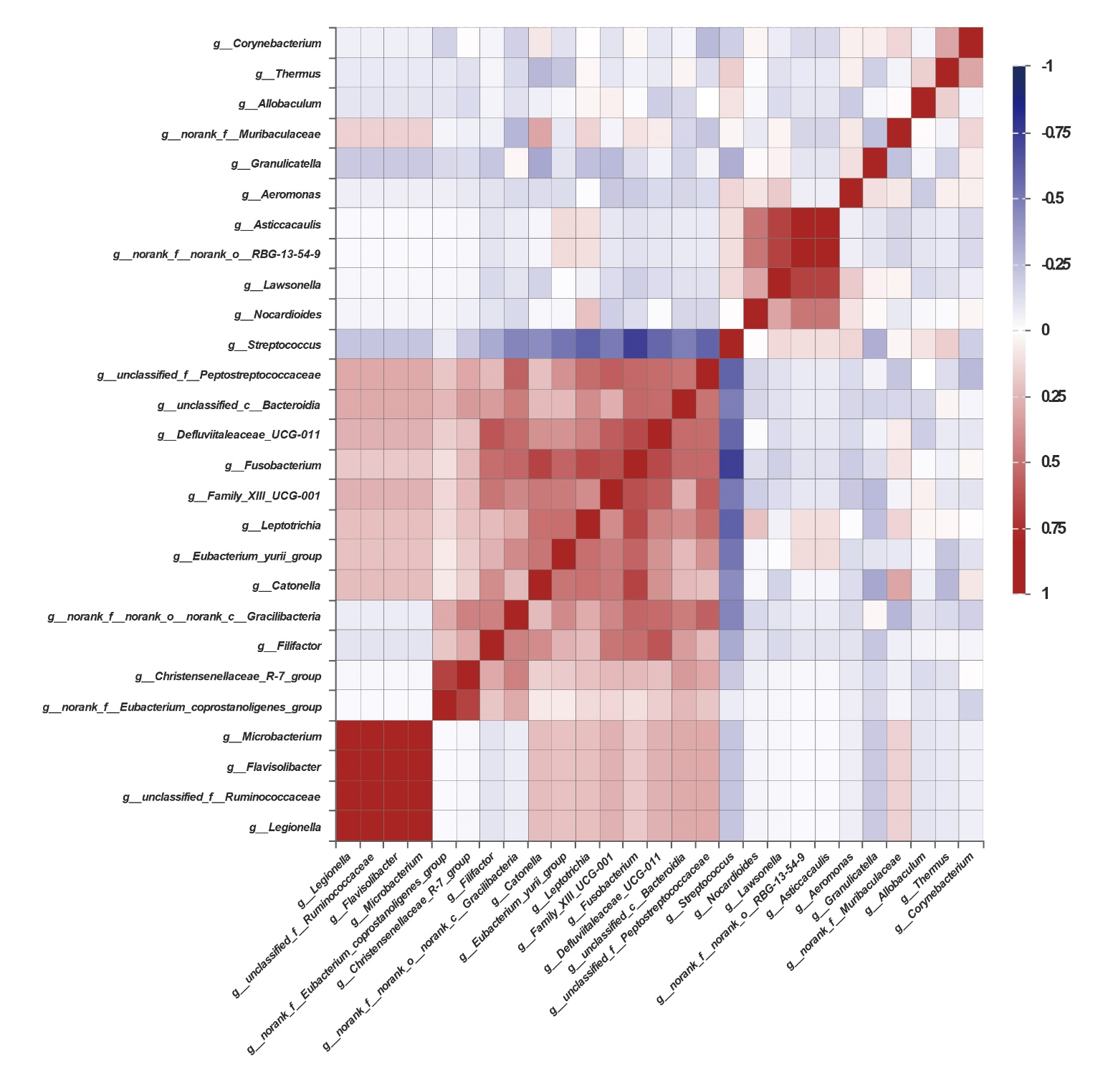


**Figure S6** Heatmaps of Spearman correlation analysis between the dominant genus. Red squares indicate positive correlations, whereas blue squares indicate negative correlations.

**
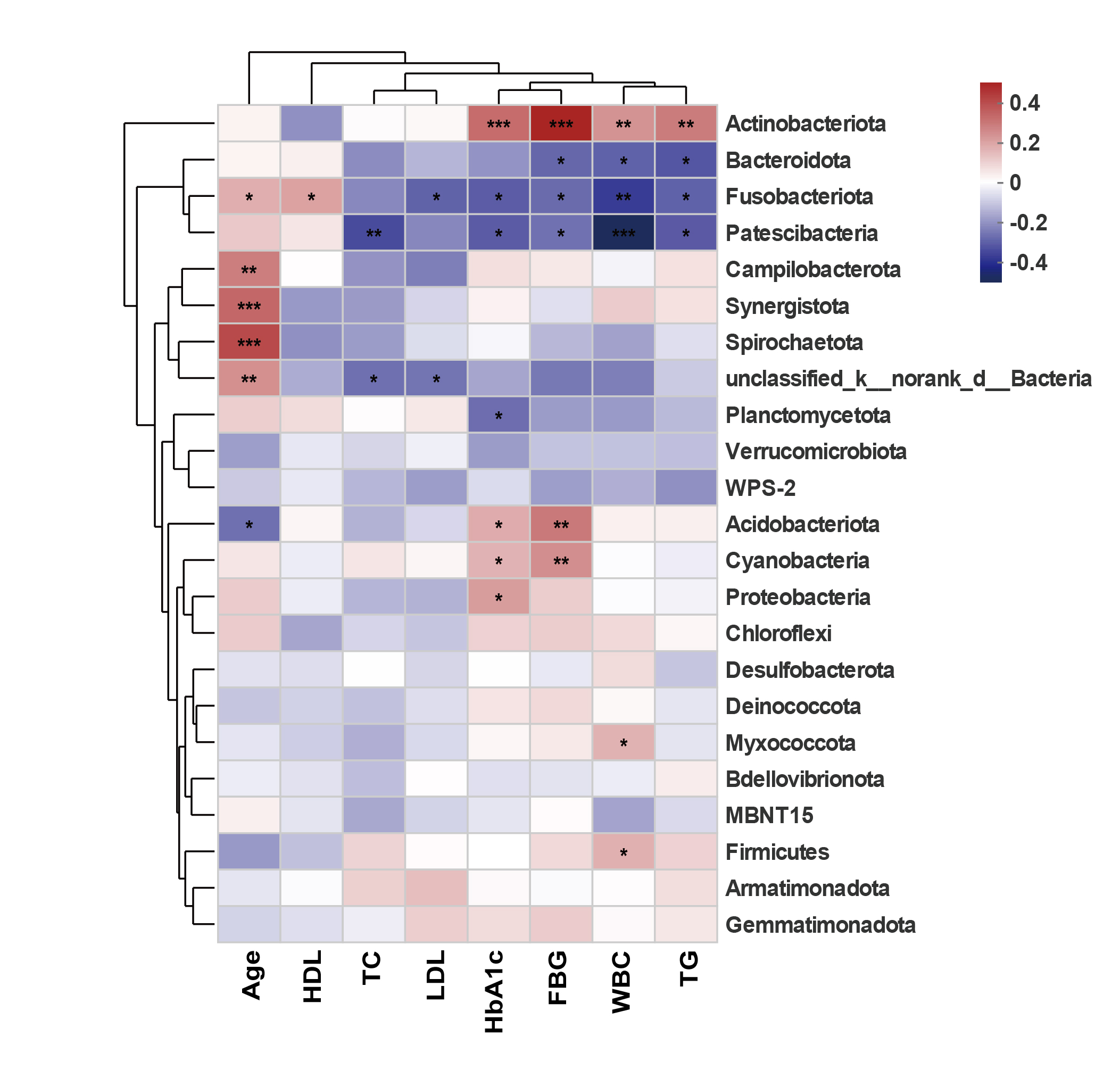
**

**Figure S7** Heatmap of the Spearman’s correlation between clinical indices and dominant phylum. Red squares indicate positive correlations, whereas blue squares indicate negative correlations. *P < 0.05, **P < 0.01, ***P < 0.001.
